# Comment: Prolonged critical avalanche burial for nearly 23 h with severe hypothermia and severe frostbite with good recovery: a case report

**DOI:** 10.1186/s13049-025-01366-7

**Published:** 2025-03-21

**Authors:** Ian J. Cohen

**Affiliations:** 1 The Rina Zaizov Department of Pediatric Hematology Oncology, The Schneider Children’s Hospital of Israel,, Petah Tikva, Israel; 2 The Sackler Faculty of Medicine, Tel Aviv University, Ramat Aviv, Israel; 3 139 Shir Hashirim St, Elkana, 44814 Israel

The survival of a 53-year-old skier, reported by Gruber et al. [1] who was buried for nearly 23 h in an avalanche (with a hypothermic duration of 27 h) is even more remarkable than it seems on first sight and raises the question if there was an additional unappreciated reason for his survival apart for the excellent treatment he received. The analysis by Eidenbebz et al. [2] of survival probability in avalanche victims after long burial (> 60 min) includes only 2 patients who survived after 10 and 17 h, none reported for more than 24 h. Even though he had an avalanche transducer, was buried only a meter below the surface and had an air pocket (and subsequently a connection to outside) he would not have been expected to survive despite having no cardiac arrest arrythmia [2]. His had an epitympanically measured temperature of 23.1^0^C (recently shown to be accurate at this temperature range [3]) a Glasgow Coma Scoree of 11and a bradycardia of 40–50 beats /min.

The initial part of the report is typical of those patients reported by Pasquier et al. [4] who had deep accidental hypothermia with core temperature below 24^0^C with vital signs.

Survival beyond 24 h of hypothermic duration has not considered worth looking for in victims of avalanche disasters [2] and after this period he would have been expected to die from delayed rewarming thrombocytopenia (DRT) [5], the complication of prolonged hypothermic duration (PHD) [5], that occurs after 24 h of hypothermia due to the build up by this time of ADP in the blood [6, 7] when the second stage of platelet aggregation, blocked below 32^0^C reappears [8].

It is also relevant that there is no mention in the report of thrombocytopenia that although potentially reversible is a central factor in mammalian hypothermia [9].

A suggested reason for the unusual, clinical picture mentioned above, reported by Pasquier [4] has been Alcohol [10] or other pharmaceutical agents such as barbiturates that have been shown not only to prevent thrombocytopenia during hypothermia [9] but also to prevent DRT.

It is suggested that if the victim in this case had taken Alcohol, or another drug such as aspirin that blocks the second phase of platelet aggregation the mystery would be solved.

## Data Availability

No datasets were generated or analysed during the current study.
